# Nutritional Value and Preventive Role of *Nigella sativa* L. and Its Main Component Thymoquinone in Cancer: An Evidenced-Based Review of Preclinical and Clinical Studies

**DOI:** 10.3390/molecules26082108

**Published:** 2021-04-07

**Authors:** Johura Ansary, Francesca Giampieri, Tamara Y. Forbes-Hernandez, Lucia Regolo, Denise Quinzi, Santos Gracia Villar, Eduardo Garcia Villena, Kilian Tutusaus Pifarre, José M. Alvarez-Suarez, Maurizio Battino, Danila Cianciosi

**Affiliations:** 1 Department of Clinical Sciences, Polytechnic University of Marche, 60131 Ancona, Italy; nubansary@gmail.com (J.A.); f.giampieri@univpm.it (F.G.); luciaregolo@gmail.com (L.R.); denise81quinzi@gmail.com (D.Q.); 2 Department of Biochemistry, Faculty of Sciences, King Abdulaziz University, Jeddah 21589, Saudi Arabia; 3 Nutrition and Food Science Group, Department of Analytical and Food Chemistry, CITACA, CACTI, University of Vigo, 36310 Vigo, Spain; tforbes@uvigo.es; 4 Research Center for Foods, Nutritional Biochemistry and Health, Universidad Europea del Atlántico, Isabel Torres 21, 39011 Santander, Spain; santos.gracia@uneatlantico.es (S.G.V.); eduardo.garcia@uneatlantico.es (E.G.V.); kilian.tutusaus@uneatlantico.es (K.T.P.); 5 Research Center for Foods, Nutritional Biochemistry and Health, Universidad Internacional Iberoamericana, Campeche 24560, Mexico; 6 Departamento de Ingeniería en Alimentos, Colegio de Ciencias e Ingenierías, Universidad San Francisco de Quito, Quito 170157, Ecuador; 7 King Fahd Medical Research Center, King Abdulaziz University, Jeddah 21589, Saudi Arabia; 8 International Research Center for Food Nutrition and Safety, Jiangsu University, Zhenjiang 212013, China

**Keywords:** *Nigella sativa*, nutritional composition, anticancer properties, molecular pathways, combined therapy

## Abstract

In recent times, scientific attention has been paid to different foods and their bioactive components for the ability to inhibit the onset and progress of different types of cancer. *Nigella sativa* extract, powder and seed oil and its main components, thymoquinone and α-hederin, have showed potent anticancer and chemosensitizing effects against various types of cancer, such as liver, colon, breast, renal, cervical, lung, ovarian, pancreatic, prostate and skin tumors, through the modulation of various molecular signaling pathways. Herein, the purpose of this review was to highlight the anticancer activity of *Nigella sativa* and it constitutes, focusing on different in vitro, in vivo and clinical studies and projects, in order to underline their antiproliferative, proapoptotic, cytotoxic and antimetastatic effects. Particular attention has been also given to the synergistic effect of *Nigella sativa* and it constitutes with chemotherapeutic drugs, and to the synthesized analogs of thymoquinone that seem to enhance the chemo-sensitizing potential. This review could be a useful step towards new research on *N. sativa* and cancer, to include this plant in the dietary treatments in support to conventional therapies, for the best achievement of therapeutic goals.

## 1. Introduction

Cancer is one of the major threats of modern life, being the second cause of death after myocardial infarction worldwide [1,2]. The mechanisms underlying cancer development and progression vary widely among cancer types and unfortunately are not well understood. However, mutations in genetic or epigenetic pathways, including tumor suppressor genes and oncogenes, have been reported in a great number of cancer cases [3]. In recent years, manifold strategies have been developed to inhibit cancer progression, including hormonal therapy, surgery, radiation and chemotherapy. Chemotherapy is one of the main approaches to treat cancer, but unfortunately in various types of cancer it has showed limited potential in long-term treatment, due to toxic effects, drug resistance and the ability of cancer cells to alter different signaling pathways to escape from death [4,5]. Indeed, tumor cells execute various approach to block the efficacy of drugs, such as the increase of drug efflux, the modification of drug metabolism, the activation of cell survival pathway, the inhibition of apoptosis and the mutation of drug targets [4,6]. In addition, the drug resistance has increased for the presence of cancer stem cells (CSCs), which have brought harmful effects against cancer treatment [7]. Besides, cancer diagnosis and management and available cytotoxic drugs are not easily affordable or available in certain places, especially in developing countries. For these reasons, a large proportion of the population prefers to patronize complementary and alternative medicine [8]. In this scenario, it is important to identify novel compounds that are non-toxic and can be used as combined therapy to improve the response of cancer cells to chemotherapeutic mediators.

Natural compounds are non-nutritive secondary plant compounds with health-promoting and disease preventive properties, mainly found in fruits, vegetables, grain, herbs, spice and other plant foods. Among them, the use of herbals in medicine is widespread and is growing dramatically because of their nourishing and synergistic actions that make them an excellent treatment choice. Among others, *Nigella sativa* (*N. sativa*) can be a valid tool for health promotion, due to its low toxicity and multiple mechanisms of action [5]. For example, recent reports suggest that the oil and extracts of *N. sativa* have antimicrobial and anti-inflammatory activity, immune stimulant properties, together with anticancer, antioxidant, hypoglycemic, spasmolytic and bronchodilator capacities [2,5,9,10,11,12,13,14,15]. Similarly, thymoquinone (TQ), the prominent compound of *N. sativa*, has shown to exert antiproliferative effects on different cancer cell lines, such as ovarian, colon, larynx, breast, leukemia, lung and osteosarcoma [11].

**Figure 1:**
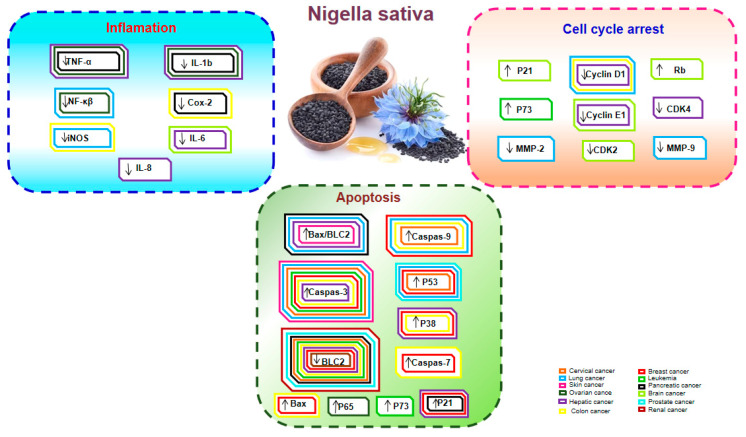
A brief overview of several cellular signaling pathways influenced by *N. sativa* and its main components through molecular targets in various types of cancer.

Recently, increased attention and intensive research efforts have been also devoted toward understanding the molecular mechanisms involved in the potent anticancer activities of *N. sativa* and its main compounds (Figure 1).

The aim of this review is to summarize the proximal, nutritional and phytochemical composition of *N. sativa*, the anticancer effect of whole extracts, its constituents and their analogs on different type of cancer together with the combined therapy with different anticancer drugs.

## 2. Literature Search

The literature search was conducted using various scientific databases, including Med-line, Scopus, Google Scholar and Web of Science, by using keywords such as “black seed”, “*N. sativa*” and “active compounds”, “*N. sativa* and nutritional composition”, “*N. sativa* and fatty acid profile”, ”*N. sativa* and analog”, “*N. sativa*” or “Thymoquinone” and “breast cancer”, ”hepatic cancer”, “colon cancer”, “renal cancer”, “cervical cancer”, “leukemia”, “lung cancer”, “ovarian cancer”, “pancreatic cancer”, “prostate cancer”, “skin cancer”, etc., “black cumin”, or “*N. sativa*” and “combine therapy” and related clinical trials. All the articles included were published from 2000 to 2020 in English.

## 3. *N. sativa* and Its Major Constituents 

*N. sativa* is an annual herb belonging to the family of Ranunculaceae. Its seeds and oil have been commonly used as a traditional remedy for a variety of health issues for more than 2000 years. Greenish, H.G., reported for the first time the chemical composition of *N. sativa* seeds in 1880, highlighting the presence of fibers, carbohydrates, proteins, oils, ashes, moisturizers, etc., as shown in Table 1 [12,13,14,15,16,17,18,19,20,21,22,23,24,25,26,27,28,29,30]. 

**Figure 2:**
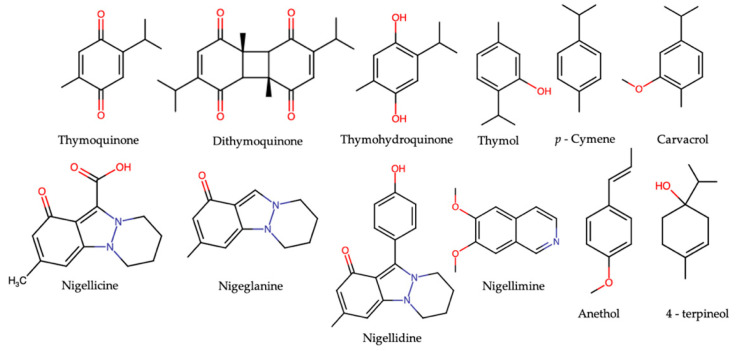
Some main components of *N. sativa*.

Diverse active compounds have been isolated and identified in different *N. sativa* varieties: beyond TQ, the main components are dithymoquinone, thymohydroquinone and thymol, p-cymene nigeglanine, nigellicine, nigellidine, nigellimine, t-anethol and 4-terpineol (Figure 2), together with carbohydrates, vitamins, mineral elements, proteins and essential amino acids [12,13,14,15,16,17,18,19,20,21,22,23,24,25,26,27,28,29,30].

*N. sativa* is also a very good source of fatty acids (Table 2), being the linoleic and oleic acids the most representative ones [14,15,16,17,18,19,20,21,22,23,24,25,26,27,28,29,30,31,32,33].

## 4. *N. sativa* as an Anticancer Agent

**Figure 3:**
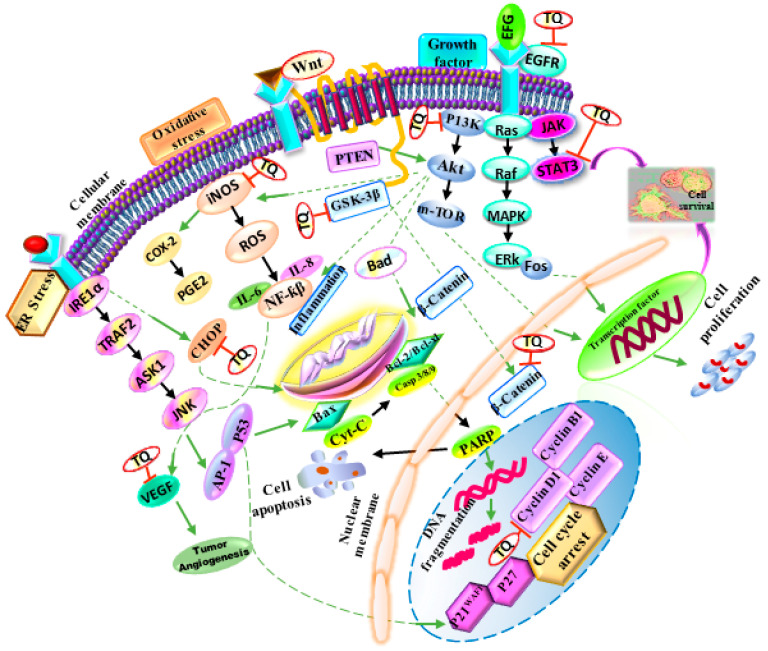
Schematic representation of the anticancer mechanisms of *N. sativa* and its main components. Thymoquinone (TQ) is able to inhibit cancer development and to regulate various genes involved in survival, proliferation, invasion, angiogenesis, metastasis and apoptosis.

*N. sativa* exerts significant antiproliferative effects against various types of cancer, such as liver, colon, breast, cervical, lung, pancreatic, prostate cancer, etc. (Figure 3). 

Several in vitro and in vivo research have revealed that its anticancer activity is mediated through the modulation of different biological processes and pathways, such as proliferation, cell cycle, apoptosis, angiogenesis, carcinogenesis and metastasis.

### 4.1. Breast Cancer

About 1.7 million women have been diagnosed with breast cancer in 2012 and over 508,000 women died due to this disease in 2011. In clinical practice, many options are available for breast cancer treatment, such as radiation, surgery, hormonal, chemotherapy and targeted therapy [34]. For example, *N. sativa* seed proteins and TQ showed antiproliferative effects, by promoting apoptosis through p38 phosphorylation, ROS production and by activating caspases, p53, p21 and Bax and decreasing Bcl-2 in human adeno- and ductal carcinoma cells, including MCF-7, 47D and MDA-MB-468 cells [35,36,37]. Furthermore, in human adenocarcinoma MDA-MB-231 triple negative cells, TQ inhibited the overexpression of chemokine receptor type 4 (CXCR4), by downregulating NF-kβ, decreased cell invasion and migration, reduced the expression of Bcl-2, Bcl-xL and survivin and activated the peroxisome proliferator-activated receptors (PPAR)-γ pathway [38,39]. Similarly, TQ inhibited tumor growth by downregulating the expression of N-Cadherin and upregulating the expression E-cadherin gene, decreasing metastatic process in mouse breast cancer cell line 4T1 [40].

Moreover, TQ was effective in damaging mitochondrial membrane integrity, by promoting the release of cytochrome c and the induction of apoptosis, arresting the cell cycle at G1 phase and concomitantly suppressing the growth of p53-deficient breast carcinoma MDA-MB-231 and MDA-MB-468 cells without affecting normal mammary epithelial cells [41]. Furthermore, in human adenocarcinoma MCF-7 cells, TQ induced apoptosis, by disrupting mitochondrial membrane potential and activating caspases and PARP cleavage and increasing Bax/Bcl2 ratio, arrested cell cycle at G2/M and sub-G1 phase via the modulation of the Akt/PTEN axis [42]. 

Regarding in vivo models, in mouse injected with MDA-MB-231 cells, TQ treatment significantly suppressed multiple lung, brain and bone metastases in a dose-dependent manner, reducing the osteolytic lesions and the expression of metastatic biomarkers [39], while in Sprague-Dawley rats injected with 7,12-Dimethylbenzathracene (DMBA) the emulsion of *N. sativa* oil fraction exhibited antiproliferative effects, by reducing the level of detrimental oxidative stress, destroying endocrine derangement and increasing apoptosis [43].

### 4.2. Colon Cancer

Colorectal cancer (CRC) is the second most common cancer in Europe and its onset is strictly correlated with several risk factors, such as high-fat diet poor in fiber, high alcohol consumption, red meat, obesity, smoking, lack of physical exercise, diabetes, older age and several genetic and epigenetic alterations. Protective effects have been found for *N. sativa* and its compounds in CRC. For instance, α-hederin and TQ, the two principal bioactive constituents of *N. sativa*, suppressed cellular viability in human colorectal adenocarcinoma HT-29 cells in a dose and time-dependent manner, by inducing apoptosis and necrosis [44]. TQ has demonstrated the efficacy also to promote autophagy and apoptosis in an irinotecan-resistant (CPT-11-R) LoVo colon cancer cells by inducing mitochondrial outer membrane permeabilization, activating JNK and p38 and inhibiting NF-ƙβ, ERK1/2 and PI3K pathways [45,46]. In addition, it promoted ROS-dependent apoptosis in human colorectal carcinoma Caco-2, HCT-116, LoVo and HT-29 cells, by activating caspases 3/7, JNK and ERKs, without showing cytotoxic effect to normal human intestinal FHs74Int cells [47]. Furthermore, high dose of TQ exerted antiproliferative effect in human colon adenocarcinoma LoVo cells through the downregulation of p-PI3K, p-Akt, p-GSK3β and β-catenin, and thereby of COX-2 and prostaglandin E2 (PGE2) [48]. Moreover, TQ mediated apoptosis in human colon adenocarcinoma HCT116 cells by increasing Bax, decreasing Bcl-2 and Bcl-xl expression and activating caspase-9, -7 and -3 and PARP cleavage, and inhibited STAT3 pathway and its downstream targets, including survivin, cyclin D1 and D2, through the suppression of JAK2- and Src-mediated phosphorylation of EGFR tyrosine kinase [49].

Regarding in vivo studies, in azoxymethane (AOM)-treated Sprague-Dawley rats, *N. sativa* and its main constituents (selenium, TQ and DL-α-tocopherol) prevented oxidative DNA damage and inhibited malondialdehyde (MDA) liver contents [50], while in PGE2-treated nude mice TQ inhibited the growth of metastatic LoVo cells by downregulating COX-2 and β-catenin levels in different tissues [48]; finally, in 1,2-dimethylhydrazine (DMH)-treated rats TQ showed antitumor effect by reducing cell proliferation, tumor volume, progression and invasion through the suppression of the colonic proliferating cell nuclear antigen (PCNA) expression and vascular endothelial growth factor (VEGF) production [51].

### 4.3. Hepatic Cancer

Hepatocellular carcinoma is the most common primary liver cancer and numerous strategies have been used for the treatment of this disease. *N. sativa* and TQ have strong antioxidant properties and have been found to exert chemopreventive effects in this kind of cancer. For example, in human hepatocellular carcinoma HepG2 cells alcoholic extracts of *N. sativa* decreased cell viability and increased apoptotic cell death through the improvement of the antioxidant condition [52]. Similarly, in HepG2, TQ arrested cell cycle at the G2/M phase and induced apoptosis, by increasing the ratio of Bax/BCL-2 through the activation of caspase-3 and -9 and by suppressing the Bcl-2 expression and PARP cleavage; additionally, it inhibited the expression of NF-ƙβ, IL-8 and its receptors, stimulated TRAIL death receptors and reduced Bcl-xS gene expression [53,54]. TQ also showed a significant inhibitory effect on phase I CYP1A1 enzyme and increased the content of glutathione (GSH) and the activity of glutathione transferase (GST) in HepG2 cells [53]. Moreover, thymol, another bioactive compound of *N. sativa*, exerted cytotoxic effect in HepG2 cells and together with carvacrol, it protected liver cells via antioxidant activity and repression of proinflammatory cytokines, such as TNF-α and IL-1β [55].

Regarding in vivo studies, in diethylnitrosamine (DENA)-treated rats *N. sativa* and TQ suppressed the EGFR/ERK1/2 signaling pathway and augmented ERK and p38 phosphorylation by modulating PCNA, c-fos and Bcl-2, and protected liver from oxidative stress injury by enhancing the levels of antioxidant enzymes, and decreasing the concentrations of alkaline phosphatase, alanine trans aminase, total bilirubin and total NOx [56,57]. Interestingly, *N. sativa* ethanolic extract exerted antitumor effect via reducing the serum levels of alpha-fetoprotein, TNF-α, IL-6 levels NO and iNOS activity in DENA-treated rats [58].

Finally, in N-Nitrosodiethylamine (NDEA) treated rats, TQ exerted antiproliferative activities and inhibited hepatic tumorigenesis by regulating cell cycle at the G1/S phase and modulating some specific proteins (i.e., p21(WAF1/CIP1, Cyclin D1), CDK4 and Cyclin E) [59].

### 4.4. Lung Cancer

The rate of mortality associated with lung cancer is high in the world. The natural source has recently received much attention for their chemotherapeutic effects against this type of cancer. In this context, in human lung carcinoma A549, NCI-H460 and NCI-H146 cells, TQ promoted apoptosis, by elevating Bax/Bcl-2 ratio, p53 and caspase 3 and 9, arrested cell cycle and suppressed cell viability, invasion and migration, by phosphorylating of ERK1/2 and suppressing cyclin D1, PCNA, MMP2 and MMP9 expression, and inhibited neoangiogenesis by decreasing proangiogenic cytokines ENA-78, Gro-alpha [60,61,62]. Interestingly, TQ had no antiproliferative effects on pulmonary fibroblast cell line MRC-5 [60]. TQ may act also as a new microtubule-targeting agent, by reducing cell viability, depolymerizing microtubules, inducing apoptosis and cell cycle arrest at the G2/M phase in A549 lung cancer cells without affecting the microtubule network of normal human umbilical vein endothelial (HUVEC) cells; it was also able to suppress tubulin polymerization in the cell-free system in a time-dependent manner and showed antimitotic effect through the bind to the tubulin in the microtubule network [63].

To the best of our knowledge, there is only one in vivo study on TQ in lung cancer: in monocrotaline (MCT) treated rats, the intake of TQ significantly increased cancer apoptosis and inhibited pulmonary arterial remodeling via p38MAPK/NF-κβ signaling pathway [64].

### 4.5. Pancreatic Cancer

Pancreatic cancer is one of the main causes of death among male cancer in the world. TQ is a potential candidate for the development of novel therapies against this cancer. For example, in human adenocarcinoma FG/COLO357 and CD18/HPAF81 cells, TQ was able to promote apoptosis, decrease motility and migration by reducing MUC4 and Bcl-xL expression via the activation of c-Jun NH2-terminal kinase and p38 pathways [65], while in human pancreatic carcinoma HS766T cells TQ from *N. sativa* oil extract induced a antiproliferative effect, by upregulating p21 expression, suppressing histone deacetylase activity, decreasing MCP-1, TNF-α, IL-1b and Cox-2 via NF-kβ modulation [66]. Additionally, in different human pancreatic cancer cells (MiaPaCa-2, BxPC-3, AsPC-1 and HPAC), TQ-4A1, TQ-5A1 and TQ-2G analogs showed more potent antiproliferative activity than parental TQ by reducing cell viability, stimulating apoptosis through the downregulation of Bcl-2, survivin and upregulation of Bax/Bcl-2 ratio and reducing COX-1 and COX-2 enzyme activity without any toxic effects on the normal cell [67,68].

### 4.6. Cervical Cancer

*N. sativa* seed extracts and TQ have shown to exert antitumor activities against cervical cancer. For example, in human cervical adenocarcinoma HeLa cells the extract of different solvents (n-hexane, methanolic and chloroform) mediated apoptosis via increasing p53, caspase-3, -8 and -9, and downregulating Bcl-2 and Bcl-X_L_ expression [69], while TQ induced apoptosis by modulating 84 genes, including the proapoptotic ones, such as BIK, FASL, BCL2L10 and CASP1, and the antiapoptotic ones, such as NF-ƙβ and RELA [70]. Interestingly, in human cervical carcinoma SiHa cells TQ induced apoptosis, arrested cell cycle at the sub-G1 phase, by modulating p53 and Bcl2 expression, decreased proliferation, reduced GSH levels and increased MDA concentration, without cytotoxic effect on normal cells (3T3-L1 and Vero cells) [71,72], while in human cervical carcinoma C33A cells TQ promoted apoptosis by activating caspase 3 [73]. Furthermore, TQ exerted cytotoxic effects, inhibited the migration and invasion processes and induced apoptosis in a time and dose-dependent manner in human cervical carcinoma CaSki cells, where it acted on Twist1 and Zeb1 and promoted E-cadherin expression [74]. Recently, the effect of TQ derivatives has been evaluated as anticancer drugs in cervical tumor. For example, Poloxin, a synthetic TQ derivative, was able to suppress serine/threonine kinase Polo-like kinase 1 (Plk1) and inhibit its functions in vitro, promoting Plk1 mislocalization, chromosome congression defects, mitotic arrest and apoptosis in HeLa cells [75].

### 4.7. Leukemia and Blood Cancer

*N. sativa* and TQ exert anticancer activities also on blood tumors. For example, in immortalized line of human T lymphocytes (Jurkat cells), and in an acute lymphocyte leukemic cell line (CEMss cells) TQ played proapoptotic activity via downregulation of ubiquitin-like, containing PHD and RING finger domains, 1 (UHRF1) and cyclic nucleotide phosphodiesterase (PDE)1A, arrested cell cycle by targeting p73-dependent pathway and stimulated caspases 3 and 8, Bax, DNA fragmentation, ROS generation and reduced Bcl-2 through mitochondrial-induced apoptosis [76,77]. Similarly, in Jurkat cells, *N. sativa* seed oil has shown anticancer activities by degrading α/β tubulin with the upregulation of the tumor suppressor p73 and by inducing apoptosis, but had no effect on α/β tubulin protein expression in normal human fibroblast cells [78]. Moreover, in different human leukemia cells, including Kasumi-1, MV4-11, THP-1 and ML-1 cells, TQ inhibited DNA (cytosine-5)-methyltransferase 1 (DNMT1) methylation activity via breakdown of the Sp1/NF-kB complex from the DNMT1 promoter, reduced colony formation and promoted cell apoptosis through activation of caspases [79]. TQ modulated also morphological patterns of apoptosis, by promoting cytoplasmic shrinkage, membrane blebbing and DNA fragmentation on murine leukemia WEHI-3 cells, thus inducing cytotoxicity [80].

A recent study also suggested that TQ could be used as an epigenetic drug that induces both histone post-translational modifications and DNA methylation regulating several tumor suppressor genes (TSGs) with subsequent apoptosis. Specifically, in Jurkat cells TQ suppressed cell growth, by upregulating some TSGs, that are usually suppressed in cancer, such as DLC1, PPAR, ST7, FOXO6, TET2, CYP1B1, SALL4 and DDIT3 along with activating several proapoptotic genes, such as RASL11B, RASD1, GNG3, BAD and BIK [81]. Moreover, in IL-6–dependent human MDN, XG-2 cell lines, TQ showed chemopreventive and chemotherapeutic effects through the inhibition of CXCL12 mediated chemotaxis and the downregulation of CXCR4 expression and increased CD95 levels and susceptibility to Fas-mediated apoptosis in primary cells isolated from multiple myeloma patients without inducing any apoptotic effect on peripheral blood mononuclear cells from healthy donors [82].

In addition, TQ derivatives, bound to terpene residues, showed more potent antiproliferative effect than the native compound through the induction of apoptosis associated with DNA laddering, the reduction of mitochondrial membrane potential and the raise in ROS in human HL-60 leukemia cells [83]. Similar effects have been obtained in in vivo studies, where TQ decreased splenomegaly and suppressed leukemia cell growth in lungs and livers after i.v. administration of TQ in leukemia-bearing mice [79], and in BALB/c mice where it reduced spleen and liver weight and suppressed WEHI-3 growth, showing interesting antileukemic activity [80].

### 4.8. Kidney and Bladder Cancer

Recent studies suggested that TQ is a potential anticancer agent in renal cancer, exerting an antiproliferative effect by (i) reducing angiogenesis, (ii) preventing interaction between HSP90 and HIF-1 α, (iii) degrading HIF-1 α protein, (iv) changing glucose, lactate, and ATP levels as a result of anaerobic metabolic disturbance, (v) decreasing mitochondrial membrane potential and consequent cytochrome c release into cytoplasm, (vi) arresting cell cycle at sub-G1, (vii) inducing ROS mediated apoptosis through the modulation of the expression of antiapoptotic proteins such as Bcl-2 and c-FLIP, or and pro-oncogenic JAK2/STAT3 pathway and (viii) preventing migration, invasion and epithelial–mesenchymal transition (EMT) via autophagy-dependent AMPK/mTOR signaling pathway, in several human renal carcinoma cells, such as Caki-1, Caki-2, A498, 786-O and ACHN cells [84,85,86]. Additionally, in human renal adenocarcinoma 769-P and 786-O cells, TQ increased the level of E-cadherin and decreased those of ZEB1, Snail and vimentin, by increasing the expression of LKB and AMPK: these results highlighted that TQ markedly reduced the metastatic phenotype and reversed the EMT [87].

Regarding bladder cancer, the total and fraction extracts of *N. sativa* were effective in inducing significant morphological changes in human renal adenocarcinoma ACHN cells but not in normal renal epithelial GP-293 cells, stimulating apoptosis and decreasing cell viability in a time and dose dependent manner [88]. Similarly, TQ showed an anticancer effect on human bladder carcinoma T24, 253J and HTB-9 cells, by inducing apoptosis through the promotion of mitochondrial dysfunction and endoplasmic reticulum stress, and by inhibiting EMT via modulation of mTOR signaling, slug, snail, N-cadherin, β-catenin and Wnt/β-catenin expressions [89,90,91]; interestingly, TQ showed no effect on normal cells (human dermal fibroblasts) [89].

To the best of our knowledge, two in vivo studies were performed on mice: the first highlighted the anticarcinogenic mechanism of TQ in tumor xenograft nude mice by blocking EMT through the downregulation of N-cadherin, β-catenin and vimentin proteins and suppressed lung metastasis [90], the latter showed the ability of TQ in inducing apoptosis through pro-oxidant effects in tumor xenograft nude mice [86].

### 4.9. Skin Cancer

There are three main types of skin cancer: squamous cell carcinoma, basal cell carcinoma and melanoma, whose epidemiological characteristics, clinical classifications and treatment methods are different [92]. Since skin cancer is frequently resistant to radiotherapy and chemotherapy, the development of cost-effective, novel and efficient treatment methods is urgently needed. Recently, natural compounds have showed potential activities against skin cancer [93]. In this context, in human melanoma MDA-MB-435 cells and in epidermoid carcinoma A431 cells TQ reduced cell viability, promoted apoptosis and cell cycle arrest, suppressed cell proliferation by upregulating the Bax/Bcl-2 ratio, caspases and cleavage of PPAR and downregulating Akt and c-Jun N-terminal kinase (JNK) phosphorylation, and induced chromatin condensation, DNA fragmentation [40,94]; on the contrary, in human keratinocyte cell line HaCaT TQ treatment showed no cytotoxic and apoptotic effect, and morphological change [94]. In addition, TQ, the conjugates of TQ with various sesquiterpenes, monoterpenes and triterpene betulinic acid showed antiproliferative activities via ROS mediated apoptosis associated with DNA laddering on 518A2 melanoma cells [83].

### 4.10. Ovarian Cancer

The most common cause of death from gynecologic malignancies is ovarian cancer since many women with epithelial ovarian cancers are diagnosed with advanced, metastatic disease characterized by carcinomatosis and abdominal ascites. In the last decade, TQ has showed an antiproliferative effect in in vitro and in vivo models against ovarian cancer. In murine ID8-NGL ovarian cancer cells, treatment with TQ decreased cell proliferation with the induction of apoptosis by increasing p-p65 and reducing Ki67, PCNA and break downing PARP; it also suppressed the NF-κβ pathway and the expression of TNF-α and IL-1β [95]. Moreover, TQ analogs showed a significant antiproliferative effect on human adenocarcinoma OVCAR-8 and CIS-A2780 cells more than the patent parent molecule [96]. 

Regarding in vivo studies, TQ has been shown to suppress tumor cell growth in a mouse model of ovarian cancer cells by inhibiting NF-κβ activation [97], while in a syngeneic mouse model of ovarian cancer (ID8-NGL mouse C57BL/6 cells) the efficacy of TQ was limited in the protumorigenic microenvironment, highlighting the need of prolonged TQ treatment (>30 days) as a harmful factor for its efficacy [95]. 

### 4.11. Prostate Cancer

Prostate cancer is the most common cancer in men and the range of clinical behavior varies from a microscopic, well-differentiated tumor to an aggressive, invasive cancer, which finally results in metastases. TQ is a potential candidate that regulates several major signaling pathways and crucial oncogenic molecules that play a central role in prostate cancer initiation, progression, invasion, metastasis and angiogenesis stage. For example, it turned EMT by raising E-cadherin expression and reducing vimentin and Slug expression, via downregulation of the TGF-β/Smad2/3 signaling pathway, and promoted apoptosis by modulating PI3K-AKT pathway in human prostate carcinoma DU145 and PC3 cells [98,99]. In human prostate carcinoma LnCaP cells, TQ deceased cell viability and increased apoptosis by activating caspase-9 [100], and inhibited the growth of human prostate C4-2B cancer cells with the activation of JNK and growth arrest and DNA damage inducible gene (GADD45a), upregulating induce apoptosis factor-1 and decreasing Bc12-related proteins, such as BAG-1, Bcl2, Bcl2A1, Bcl2L1 and BID [101]. Finally, TQ showed prooxidant cytotoxic mechanism by oxidative DNA damage through copper-dependent pathway via mobilizing and reducing endogenous cellular copper in different prostate cancer cell lines, including DU145, LNCaP, PC3 and C42B [102].

### 4.12. N. sativa and Other Cancers

Many in vitro and in vivo studies have also confirmed the anticancer activities of *N. sativa* and TQ in other types of cancer. For example, TQ decreased chemoresistance and angiogenesis through blocking NF-κβ activation, DNA-binding activity, survivin and VEGF and increased the expression of cleaved caspase-3 and Smac in human osteosarcoma cells SaOS-2121; these results were confirmed in an in vivo study on male athymic BALB/c nu/nu mice treated with TQ, where it was able to prevent tumor angiogenesis and inhibit osteosarcoma growth through decreasing the expression of inhibitor of apoptosis proteins, VEGF, prosurvival molecules, such as survivin and XIAP, and proliferation markers, including CD34 and Ki-67 [103]. Moreover, it suppressed osteolytic bone metastasis, such as bone marrow of the femora, tibiae, mandibles and osteolytic lesions in NCr-Foxn1nu female mice injected with breast MDA-MB-231-lucC cancer cells into the left cardiac ventricle [39]. Moreover, volatile oil of *N. sativa* has shown chemopreventive potential in fibrous histiosarcoma by decreasing malignant tumor sizes, incidences and multiplicities in carcinogens-treated rats [104].

It has been reported that TQ showed anticancer activity also in oral cancer, by downregulating p38β MAPK in oral T28 cancer cells, by increasing the expression of the proapoptotic proteins Bad and Bid and activating p53 and caspase 3 cleavage [105]. It was responsible for apoptosis and autophagy-mediated cell death in oral cancer SCC-4, SAS, SASVO3, OC2 and B S-G cells, where it modulated Bax expression and caspase-9 activation, autophagic vacuoles and LC3-II proteins by caspase activation-dependent apoptosis [106]. Regarding in vivo studies, TQ has been shown an antitumor effect in BALB/c nude mouse xenograft model by inducing apoptosis and autophagy [106] and in DMBA treated hamster buccal pouch carcinogenesis through repaired expression of cytokeratin and suppressed tumor formation [107]. 

*N. sativa* seed oil has shown anticancer activities also in brain U87 cancer cells through the degradation of α/β tubulin with the upregulation of the tumor suppressor p73 gene and induction of apoptosis [78]. In addition, it also induced cellular senescence in human malignant glioma cells U87MG, U118MG and A172 by suppressing cell cycle progression in the G1 phase, and increasing p53, p21, Rb and reduced lamin B1, cyclin E and CDK-2 [108].

Finally, TQ acted as a potent agent in the treatment of head and neck cancer by exerting antiproliferative and radio sensitizing properties through the decrease of colony formation and the induction of apoptosis in tongue carcinoma SCC25, CAL27 and HNSCC cells [109].

Table 3 summarizes the anticancer effects exerted by *N. sativa* and its main components in different types of cancers.

## 5. Combined Therapy

A wide variety of chemotherapeutic drugs have been developed for the treatment of different malignancies since the 1940s. Despite the advent of numerous highly efficient cytotoxic agents, the overall rate of cancer-related death is barely reduced during last 70 years. Several major drawbacks have been identified in anticancer chemotherapy, such as non-specific cytotoxicity, which results in bone marrow suppression and other organ toxicity and the development of drug resistance. Indeed, in aggressive malignant neoplasm, a highly variable sensitivity to therapeutics can be observed and some of cell lines can develop resistance to the treatment; the combined effects of bioactive compounds and conventional therapy should be higher compared to a single compound, slowing the development of resistance [110].

An increased drug efflux, alteration of the molecular targets, increased repair of drug-induced DNA damage, activation/suppression of signaling pathways lead to an upregulation of survival molecules and avoidance of apoptosis, the most prominent reasons of tumor cell challenge against chemotherapy [111,112]. The use of anticancer adjuvant therapy with natural bioactive molecules is known to have notable efficacy and protect from vital side effects of chemotherapeutics. For example, TQ pretreatment following Gemcitabine administration synergistically increased apoptosis and inhibited tumor growth both in vitro on human pancreatic cancer cell lines PANC-1, BxPC-3 and AsPC-1 and in vivo models on PANC-1 cells orthotopic xenograft mice by: (i) suppressing Notch1, NICD and Akt/mTOR/S6 signaling pathways, (ii) decreasing antiapoptotic proteins, such as Bcl-2, Bcl-xL and XIAP, and increasing the activation of proapoptotic molecules, including caspase-3, -9 and Bax and (iii) inhibiting the phosphorylation and nuclear translocation of p65 [113]. Furthermore, an analog of TQ induced apoptosis by downregulating Bcl-2, Bcl-xL, survivin, XIAP, COX-2 combined with Gemcitabine and oxaliplatin in Gemcitabine resistant pancreatic cancer MiaPaCa-2 cells [67,68]. Recent studies suggested that TQ with Gemcitabine led to considerable reduction of cell proliferation, induction of apoptosis, necrosis and autophagy in and pancreatic cancer PANC-1 and Mia PaCa-2 cells via increasing caspase 3, PARP cleavage, downregulating PKM2, reducing cell viability and regulating two independent microRNA (miR-101 or miR-24-2) [114]. Additionally, TQ significantly increased the antiproliferative activity of gemcitabine by depleting tumor associated resistant stem cell fraction through the induction of apoptosis by 1.5–3.6 folds and exhausted breast cancer associated stem cell (CD44(+)/CD24(−)/(low) clone in gemcitabine-resistant breast MCF-7 and T47D cells by 3.8%–27.5%, respectively [115]. It has also been reported that TQ synergistically augmented the anticancer activities of paclitaxel, cisplatin and docetaxel, enhancing apoptosis via JAK-STAT and p53 signaling network in triple-negative breast cancer [41,116]. In the case of breast tumor xenograft mice, TQ combined with doxorubicin showed high inhibition of tumor growth through enhancing p-p38 protein expression in tumor tissues, downregulating XIAP, survivin, Bcl-xL and Bcl-2; at the same time it raised catalase, SOD and glutathione levels in liver tissues [35]. 

Another recent study reported that synergistic effects of TQ and Vitamin D3 or 5-FU could control colon cancer progression in azoxymethane-induced rat model, by arresting the cell cycle in the G1 phase. Specifically, the combined treatment showed a remarkable antitumor effect by dramatically reducing tumor growth and large aberrant crypts loci via Wnt pathway, by decreasing β-catenin, NF-κβ, COX-2, iNOS, VEGF and HSP-90 and increasing DKK-1, CDNK-1A, TGF-β1, TGF-β/RII, smad4 and GPx in the early stage of CRC [117,118]. TQ with cisplatin showed also synergistic effect in lung carcinoma NCI-H460, NCI-H146 and LNM35 cells and in mouse xenograft model, by decreasing tumor mass and volume without affecting kidneys or liver [61,119].

In case of leukemia, TQ combined with doxorubicin significantly inhibited cell growth in multidrug-resistant, HL-60 and Jukart cells, by increasing Bax/Bcl-2 mRNA ratio and increasing ROS [120,121]. Moreover, in human platelets TQ and the anticancer drug ABT-737 activated protein kinase A (PKA) in a caspase-3-dependent manner, which correlated with platelet inhibition and apoptosis that significantly contributed to the bleeding risk in chemotherapy patients [122].

In bladder cancer, in adult male Wistar rat *N. sativa* oil and TQ exerted protective effect in long term cancer chemotherapy with cisplatin [123], while the combination of platinum drugs cisplatin and oxaliplatin with TQ was effective to overcome drug resistance in ovarian cancer chemotherapy in A2780 cisR cell [124]. In addition, the combined treatment of TQ and cisplatin increased apoptosis through regulating Bcl-2 and Bax genes and exerted antitumoral effects on SK-OV-3 and in ID8-NGL cell line, while in C57BL/6 mice, it inhibited NF-κβ activity and induced DNA damage and proapoptotic effects, reducing cell proliferation [95,125].

Moreover, TQ and Zoledronic combination raised the caspase 3/7 activity in prostate PC-3 cancer cells, but not in DU-145 cell line and showed also significant cytotoxic activity by DNA fragmentation and apoptosis induction [126]; interestingly when TQ was coadministrated with docetaxel, it showed therapeutic effect in DU-145 cells via increased apoptosis through PI3K and ERK signaling pathways modulation [99].

The treatment with TQ and cisplatin or diosgenin decreased cell viability and increased apoptosis, reduced cell growth and proliferation in oral squamous cell carcinoma cell line UMSCC-14C, by upregulating the expression of p53 and caspase 9 and downregulating the expression of Bcl 2 [94,127].

Finally, TQ showed synergism with radiation in the treatment of head and neck squamous cell carcinoma in SCC25 and CAL27 HNSCC lines due to its antiproliferative and radio sensitizing properties [109].

Table 4 shows the effects of the combined treatment of *N. sativa* and its constituents with anticancer drug and/or other bioactive molecule in various types of cancer.

## 6. Clinical Studies

To the best of our knowledge, few clinical studies have been performed with *N. sativa* [128,129,130]. In children (2–18 years) with acute lymphoblastic leukemia *N. saliva* seed significantly improved the treatment outcome: the combination of TQ with other cytotoxic drugs, including L-asparaginase, administered at dose 40 mg/kg 2 times orally for 6 months led to a remission rate of 92%; at the same time TQ increased survival rate and exerted non-toxic effects in healthy subjects [128]. Furthermore, in one randomized phase 2 parallel-grouped clinical trial TQ reduced lesion in patients (aged 18–75 years) suffering from oral potential premalignant lesions at dose 100/200 mg for 2 years (ClinicalTrials.gov Identifier: NCT03208790) [129]. In addition, in an Arabian Phase I trial TQ was found safe and well-tolerated in patients up to 10 mg/kg/day, but at this dosage, there was no significant anticancer activity found [130].

## 7. Conclusions and Future Perspectives

Despite the efforts, cancer still remains undefeated in the history of mankind with an annual death rate of over a million. Natural products like *N. sativa* have contributed to the discovery of new anticancer strategies and its major constituents such as TQ are promising candidates to remarkably combat cancer progression. Their effectiveness to prevent different processes related to cancer, such as proliferation, migration and invasion, angiogenesis and metastasis were here described and reported (Table 3). Moreover, we also showed that TQ enhances the anticancer effect of chemotherapeutic agents and radiotherapy in combined therapy. Results accomplished from such studies may open a new route in the field of alternative medicines for preventing various types of cancer. Hence, further investigation is required to study the anticancer effect of *N. sativa* and its constituents in suitable combinations with existing chemotherapeutic agents for effective treatment of many cancers. Moreover, it should be considered the specific cellular and molecular targets of various constituents of *N. sativa*. It is known that *N. sativa* can act both as a ROS inducer at relatively high concentration and as an antioxidant at low concentration. For well understanding these differential cellular effects, further in vivo and in silico studies should be performed at both the genomic and proteomic level, especially considering that the data obtained in vitro are generally difficult to translate in in vivo conditions as recently reported [131].

Therefore, more studies are recommended to improve the pharmacodynamics and bioavailability of *N. sativa* in order to highlight its potency and poor bioavailability. These issues can be resolved by synthesizing various analogs of *N. sativa* and its constituents and formulating those into different delivery systems in adequate preclinical and clinical studies, where *N. sativa* or its derivatives can potentiate the antitumorigenic potential of various conventional and well-established anticancer agents.

## Figures and Tables

**Table 1:** Chemical and nutritional composition of *Nigella sativa*.

**Proximate Analysis**	**(%)**
Moisture	3.8–7.0
Crude protein	18.59–31.2
Crude fat	22.0–56.4
Total ash	4.0–4.29
Crude fiber	3.7–4.7
Carbohydrates	24.9−40.0
**Fat Soluble Vitamin**	**mg/Kg**
DL-α-tocopherol	0.177
DL-β-tocopherol	9.027
DL-γ-tocopherol	5.427
All trans retinol	0.277
**Water Soluble Vitamin**	**mg/Kg**
B1	13–18
B6	4–15
Niacin	33–97
Folic acid	400–870
**Minerals**	**mg/100 g**
Iron	9.10−15.40
Copper	1.50−3.75
Sodium	41.20−55.0
Potassium	442.3−675.0
Calcium	154.4−305.0
Zinc	3.36−6.60
Phosphorus	378.12−576.90
Magnesium	134.90−147.05

**Table 2:** Fatty acid profile of *N. sativa*.

Fatty Acid Profiles	Molecular Formula	%
Myristic acid	CH_3_(CH_2_)_12_COOH	0.29–1.1
Myristoleic acid	C_14_H_26_O_2_	2.42–2.65
Palimitic acid	C_16_H_32_O_2_	9.9–18.4
Stearic acid	C_18_H_36_O_2_	1.51–3.70
Oleic acid	C_18_H_34_O_2_	18.9–25.69
Linoleic acid	C_18_H_32_O_2_	47.0–67.5
Linolenic acid	C_18_H_30_O_2_	0.19–2.70
Arachidic acid	C_20_H_40_O_2_	0.19–0.25
Eicosenoic acid	C_20_H_38_O_2_	0.32–1.0
Arachidonic acid	C_20_H_32_O_2_	0.19–0.25
Behenic acid	C_22_H_44_O_2_	1.80–2.60
Saturated fatty acids	n.a	16.25–26.7
Monounsaturated fatty acids	n.a.	19.22–29.11
Poly unsaturated fatty acids	n.a.	49.1−72.42

**Table 3:** Anticancer activities of *N. sativa* and its main component thymoquinone in different cancer types.

*N. sativa* Extracts/Fraction/ Component/Analogs	Experimental Models	Intervention	Main Results	References
**Breast cancer**				
In vitro				
Thymoquinone	MCF-7 and MDA-MB-231	40 μM for 12 h	- Decrease of cell proliferation - Induction of apoptosis - Increase of ROS	[35]
Thymoquinone	MDA-MB-468 and T-47D cells	0.01–60 μM for 12, 24 and 48 h	- Decrease of cell proliferation - Induction of apoptosis and arrest cell - cycle at G1 phase - Modulation of protein translation - of cyclin D1 - Inhibition of cell survival	[36]
Proteins from black seeds	MCF-7 cells	5–60 µg/mL for 48 h	- Inhibition of cell proliferation - Induction of apoptosis - Arrest of cell cycle	[37]
Thymoquinone	MCF-7, MDA-MB-231 and BT-474 cells	48, 40 and 32 mM, 24,14 and 11 mM, 38, 18, and 21 mM for 12, 24 and 48 h	- Inhibition of cell proliferation via - PPAR- activation pathway - Induction of apoptosis - Reduction of the migration and invasion	[38]
Thymoquinone	MCF7, MDA-MB-231, and BT-549 cells	25, 50 μM for 24 h	- Reduction of the migration and invasion - Inhibition of tumor growth and metastasization	[39]
Thymoquinone	Mouse breast cancer cell line 4T1	5 μM for 6 h	- Inhibition of cell growth, migration and invasion - Down-regulating of N-Cadherin and upregulation of E-cadherin expression	[40]
Thymoquinone	MDA-MB-231 and MDAMB-468	2.5–5 μM for 72 h	- Inhibition of cell growth - Induction of apoptosis - Arrest of cell cycle at G1 phase - Reduction of mitochondrial membrane integrity	[41]
Thymoquinone	MCF-7 cells	100 μM for 48 h	- Induction of apoptosis - Disruption of mitochondrial membrane potential - Activation of caspases and PARP cleavage - Arrest of cell cycle at G2/M and sub-G1 phase via the modulation of Akt/PTEN axis	[42]
In vivo				
Thymoquinone	NCr-Foxn1nu, female mice injected with MDA-MB-231-Luc + cells	2 mg or 4 mg/kg body weight for 4 weeks	- Suppression of bone metastasis	[39]
*N. sativa* emulsion of oil fraction	Six-week-old female Sprague-Dawley rats	400 mg/100 g for 3 months	- Increase of tumor cell loss - Reduction of oxidative damage - Decrease of endocrine derangement	[43]
**Colon cancer**				
In vitro				
α-hederin and thymoquinone	HT-29 cells	6–40 μM (Alpha) and 25–150 *μ*M (TQ) for 24, 48 and 72 h	- Decrease of cell proliferation - Induction of apoptosis and necrosis	[44]
Thymoquinone	CPT-11-R LoVo cells	2–8 μM for 24 h	- Increase of autophagic cell death - Activation of apoptosis	[45]
Thymoquinone	CPT-11-R LoVo cells	2, 4, 6, and 8 μM for 24 h	- Suppression of metastasis by NF-ƙβ inhibition and activation of JNK and p38	[46]
Thymoquinone	Caco-2, HCT-116, LoVo, DLD-1 and HT-29 cells	12.5–110 μM for 24 and 48 h	- Inhibition of cell growth - Induction of apoptosis via ROS generation	[47]
Thymoquinone	LoVo cells	5–20 μmol/L for 24 h	- Inhibition of cancer cell growth and migration	[48]
Thymoquinone	HCT116 cells	0.1 mL for 24 h, 48 h and 72 h	- Induction of apoptosis by blocking - STAT3 pathway	[49]
*N. sativa*	AOM treated male Sprague Dawley rats	200 mg/kg for 5 weeks	- Inhibition of oxidative DNA damage - Inhibition of liver MDA	[50]
Thymoquinone		0.2 mg/kg for 5 weeks		
All-trans-retinol plus		1.2 mg/kg for 5 weeks		
Selenium		100 mg/kg for 5 weeks		
DL-α-tocopherol		10 mg/kg for 5 weeks		
Thymoquinone	PGE2 treated nude mice	0.5, 10 and 20 µmol/L/ 3 time/week for three weeks	- Inhibition of metastasization - Inhibit of tumor growth	[49]
Thymoquinone	DMH treated male Albino Wistar rats	10 mg/kg/day	- Inhibition of cellular proliferation - Decrease of PCNA and VEGF - Suppression of cancer invasion	[51]
**Hepatic Cancer**				
In vitro				
Alcoholic extracts of *N. sativa*	HepG2 cells	1000, 2500, and 5000 μg/mL for 6, 24, 48, and 72 h	- Improvement of antioxidant status - Induction of apoptotic death	[52]
Thymoquinone	HepG2 cells	6–50 μM for 6, 12, 18 h	- Induction of cell cycle arrest at G2/M phase - Enhancement of TRAIL-induced cell death - Stimulation of pro-apoptotic Bcl-xS - Inhibition of cell growth	[53]
Thymoquinone	HepG2 cells	20, 40,60, 80 and 100 μM for 24, 48 and 72 h	- Arrest of cell cycle at G2/M phase - Induction of apoptosis - Decrease of VEGF	[54]
Thymol and cravacol	HepG2 cells	25, 50, and 100 mM for 24, 48, 72 h	- Improvement of oxidative stress and inflammation	[55]
In vivo				
*N. sativa* extract	DENA induced preneoplastic stage of HCC in rats	150, 250, 350 mg/kg/day body weight for 16 weeks	- Antioxidant effects - Inhibition of EGFR/ERK1/2 pathway	[56]
Thymoquinone	DENA induce male Wistar albino rats	4 mg/kg/day for 7 days	- Increase of antioxidant activity - Control of cell proliferation	[57]
N. sativa extract	DENA-induced hepatocarcinogenesis Male Wistar rats	250 mg/kg/day for 5 days	- Reduction of serum levels of alpha-fetoprotein, TNF-α, IL-6 levels NO and iNOS activity	[58]
Thymoquinone	NDEA induce male Wistar strain albino rats	20 mg/kg body weight	- Decrease of cell proliferation - Regulation of G1/S phase - cell cycle transition - Decrease of tumor markers - Inhibition of hepatic nodule formation - Reduction of tumor multiplicity	[59]
**Lung cancer**				
In vitro				
Thymoquinone	A549 cells	25, 50 and 100 μM For 72 h	- Reduction of viability - Increase of apoptotic cell death	[60]
Thymoquinone	NCI-H460 and NCI-H146 cells	20, 40, 60, 80 and 100 μM for 24 h	- Reduction of cell viability - Induction of apoptosis	[61]
Thymoquinone	A549 cells	5, 10, 20, 40, 80, 160 μmol/L for 24, 48, or 72 h	- Inhibition of metastasization - Inhibition of proliferation, migration, - and invasion via ERK1/2 pathway - Inhibition of the expression level - of PCNA, cyclin D1, MMP2, and MMP9 mRNA, P16	[62]
Thymoquinone	A549 cells	10, 25 μM for 24 h	- Induction of G2/M cell cycle arrest - and apoptosis - Depolymerization of microtubule network	[63]
In vivo				
Thymoquinone	MCT treated male Sprague–Dawley rats	8 mg/kg, 12 mg/kg, 16 mg/kg/day for 2 weeks	- Induction of apoptosis - Inhibition of pulmonary arterial remodeling - via p38MAPK/NF-κB signaling pathway	[64]
**Pancreatic cancer**				
In vitro				
Thymoquinone	MUC4 expressed FG/COLO357 and CD18/HPAF cells	10–100 μmol/L for 24 h	- Inhibition of cell growth - Downregulation of MUC4 expression - Induction of apoptosis	[65]
Thymoquinone	HS766T cells	25, 50, 75 μM for 3, 6, 24 h	- Upregulation of p21 - Suppression of histone deacetylase activity, - Decrease of MCP-1, TNF-α, IL-1b and Cox-2 via NF-kβ modulation	[66]
Thymoquinone-2G, Thymoquinone-4A1 and Thymoquinone-5A1	MiaPaCa-2, BxPC-3, AsPC-1 and HPAC	10 μM for 72 h	- Inhibition of cell growth - Induction of apoptosis and G2/M phase cell-cycle arrest - Modulation of NF-κB transcription	[67]
ATQTHB and ATQTFB	MiaPaCa-2 and BxPC-3 cells	5, 10, 25 μM for 72 h	- Decrease of cell proliferation	[68]
**Cervical cancer**				
In vitro				
Organic extracts of *N. sativa* (methanolic, n-hexane, and chloroform extracts)	HeLa cells	21.1%, 30% and 42% for 24 h	- Induction of apoptosis by the modulation - of pro- and anti-apoptotic gene	[69]
*N. sativa* oil fraction and thymoquinone	HeLa cells	0.03 to 2 μL/mL and 6.25 to 100 μM for 48 h	- Inhibition of cell proliferation - and migration - Induction of apoptosis	[70]
Thymoquinone	SiHa cells	to 30 μg/mL for 24, 48 and 72 h	- Decrease of cell viability - Arrest of cell cycle at sub-G1 phase - Promotion of apoptosis	[71,72]
Thymoquinone	SiHa and C33A cells	10–100 μM for 22 h	- Induction of apoptosis	[73]
Thymoquinone	SiHa and CaSki	1, 5, 10, 20 and 40 μM for 12, 24, 36 and 48 h	- Suppression of cell growth - Induction of apoptosis - Inhibition of migration and invasion	[74]
Poloxin	HeLa cells	5–25 μM for	- Suppression of serine/threonine kinase Polo-like kinase 1 (Plk1)	[75]
**Leukemia/Blood cancer**				
In vitro				
Thymoquinone	Jurkat cells	10 and 20 μM for 24 h	- Promotion of apoptosis	[76]
Thymoquinone	CEMss cell	50, 25, 12.5, 6, 3 and 1.5 μg/mL for 24, 48, 72 h	- Decrease of cell viability - Arrest of cell cycle at S phase - Promotion of apoptosis - Breakdown of cellular DNA	[77]
Thymoquinone	Jurkat cells	100 mM for 24h	- Degradation of α/β Tubulin	[78]
Thymoquinone	Kasumi-1, MV4–11, THP-1 and ML-1	1, 10, 30 and 300 nM, 1, 3, 10, 30 and 100 μM for 24 h	- Inhibition of cancer cell growth - Decrease of DNMT1 methylation - Decrease of colony formation - Increase of cell apoptosis	[79]
Thymoquinone	Murine WEHI-3 cells	100, 50, 25, 12.5, 6, 3 and 1.5 mg/mL for 24 h	- Decrease of cell viability - Promotion of apoptosis	[80]
Thymoquinone	Jurkat cells	5–10 μM for 24 h	- Upregulation of tumor suppressor genes	[81]
Thymoquinone	MDN and XG-2 cell lines	0.5–50 μM for 0.25–48 h	- Inhibition of CXCL12 mediated chemotaxis - Down-regulation of CXCR4 expression	[82]
Thymoquinone derivatives bound to terpene residues	Human HL-60 leukemia	5 μM for 72 h	- Decrease of cell proliferation - Induction of apoptosis associated - with DNA laddering - Increase in ROS	[83]
In vivo				
Thymoquinone	C57BL/6 mice	15 and 30 mg/kg/2 dose/week for 3 weeks	- Reversion of splenomegaly - Inhibition of leukemia cell growth in lungs and livers	[79]
Thymoquinone	BALB/c mice	100 mg/mL, 50 mg/kg for 3 weeks	- Inhibition of tumor growth	[80]
**Urinary cancer**				
In vitro				
Thymoquinone	Caki cells	25, 50 and 75 μM for 24 h	- Induction of apoptosis through downregulating c-FLIP and Bcl-2 - Increase of intracellular ROS	[84]
Thymoquinone	Caki-1, Caki-2, A498 cells	0.5–10 μM for 24, 48 h	- Induction of apoptosis - Decrease of HIF-1 protein	[85]
Thymoquinone	Caki-1 cells	1–25 μM for 24 h	- Increase of intracellular ROS - Induction of apoptosis	[86]
Thymoquinone	786-O and RCC 769-P cells	0, 10, 20, 40, 60, 80 and 100 μmol/L for 24 h or 48 h	- Inhibition of metastatic cell growth via AMPK/mTOR signaling pathway	[87]
Hydroalcoholic extract of *N. sativa* and its fraction	ACHN cells	50, 100, 250, 500, 750, 1000, 1250, 1500, 1750, and 2000 mg/mL for 24, 48 and 72 h	- Decrease of cell viability - Promotion of apoptosis	[88]
Thymoquinone	T24 and HTB-9 cells	10 to 75 μM for 48 h	- Decrease of cell viability - Inhibition of EMT by mTOR signaling	[89]
Thymoquinone	T24 and 253J bladder cancer cells	10–40 μM for 24 h	- Decrease of cell viability and proliferation - Decrease of metastasization - Reverse of EMT	[90]
Thymoquinone	T24 and 253J bladder cancer cells	40–80 μmol/L for 24 h	- Decrease of cell viability and proliferation - Induction of endoplasmic reticulum stress-mediated apoptosis	[91]
In vivo				
Thymoquinone	male BALB/c nude mice	1 mg/kg or 5 mg /kg for 3 times/day for 35 days	- Decrease of tumor volume - Induction of pro-oxidant effect	[86]
Thymoquinone	nude mice	10 mg/kg/every 3 days for 21 days	- Inhibition of EMT and metastasis	[90]
**Skin cancer**				
In vitro				
Thymoquinone	A431 cells	2–100 μM for 24 or 48 h	- Inhibition of cell proliferation - Induction of apoptosis	[94]
Thymoquinone	Melanoma MDA-MB-435 cells	5 μM for 6 h	- Inhibition of cell growth, migration, and invasion	[40]
Thymoquinone and its conjugated derivatives	518A2 melanoma	3.9 μm for 72 h	- Enhancement of anticancer activity	[83]
**Ovarian cancer**				
In vitro				
Thymoquinone	Murine ID8-NGL cells	25 μM for 24	- Decrease of cell proliferation - Induction of apoptosis by increasing p-p65 and reducing Ki67, PCNA and break downing PARP - Suppression of NF-κβ, TNF-α and IL-1β	[95]
Thymoquinone and its analogs	OVCAR-8 and CIS-A2780	10 mM for 24 h	- Induction of cytotoxic effect	[96]
In vivo				
Thymoquinone	ID8-NGL treated C57BL/6 mice	20 mg/kg thrice weekly for 10 days and 30 days	- Induction of cytotoxic effects - Induction of DNA damage	[95,97]
**Prostate cancer**				
In vitro				
Thymoquinone	DU-145 and PC3 cells	0.1–10 μM for 24 h	- Suppression of metastatic phenotype - Reverse of EMT	[98]
Thymoquinone	DU-145 cells	60 μM for 72 h	- Promotion of cytotoxicity - Induction of apoptosis	[99]
Thymoquinone	LnCaP cells	1, 5, 10, 25 and 50 μM for 24 or 48 h	- Decease of cell viability - Increase of apoptosis by activating caspase-9	[100]
Thymoquinone	C4-2B and PC-3 cells	25–150 μmol/L for 24–48 h	- Inhibition of cell growth - Increase of intracellular ROS - Decrease of GSH levels	[101]
Thymoquinone	PC3, LNCaP, DU145 and C42B cells	0–20 μM for 72 h	- Induction of oxidative DNA damage - Reduction of endogenous cellular copper	[102]
**Bone cancer**				
In vitro				
Thymoquinone	SaOS-2 cells	20, 40 and 80 μmol/l for 24 h	- Decreased of chemoresistance and angiogenesis - Inhibition of cell viability	[103]
In vivo				
Thymoquinone	Male athymic BALB/c nu/nu mice	6 mg/kg/day for 15 days	- Inhibition of tumor angiogenesis and tumor growth	[103]
Thymoquinone	MDA-MB-231-lucC expressing NCr-Foxn1nu, female mice	2 mg or 4 mg/kg for 4 weeks	- Decrease of metastatic - osteolytic lesions - Decrease of bone colonization	[39]
**Fibrous histiosarcoma**				
In vivo				
*N. sativa* and volatile oil	DMBA induced Wistar rats	1000 or 4000 ppm daily for 30 days	- Decrease of tumor size incidences and multiplicities	[104]
**Oral cancer**				
In vitro				
Thymoquinone	T28 cells and N28 cells	5–100 μM for 24 h	- Induction of apoptosis - Reduction of viability	[105]
Thymoquinone	SCC-4, SAS, SASVO3, OC2, and (B) S-G cells	20, 40, and 60 μM for 24h	- Induction of autophagic cell death - Promotion of caspase-9-dependent apoptosis	[106]
In vivo				
Thymoquinone	BALB/c AnN.CgFoxn ^nu^/Crl Narl mice	10 and 25 mg/kg body wt for 20 days	- Reduction of tumor weight and volume	[106]
Thymoquinone	DMBA induced hamster rats	30 mg/kg body wt for 14 weeks	- Potent chemo preventive efficacy - Prevention of the incidence of neoplasm and - cytokeratin expression	[107]
**Brain cancer**				
In vitro				
Thymoquinone	U87 cells	100 mM for 24 h	- Degradation of α/β Tubulin	[78]
Thymoquinone	U87MG, U118MG, and A172 cells	10–100 μM for 24 h	- Suppression of tumor growth	[108]
**Head and neck**				
In vitro				
Thymoquinone	SCC25 and CAL27 HNSCC cells	0–80 μM for 72 h	- Induction of apoptosis	[109]

**Table 4:** Combined therapy of *Nigella sativa* and its constituents with anticancer drug and/or other bioactive molecule in vitro and/or in vivo models of various types of cancer.

*N. sativa* Extracts/Fraction/ Component/Analogs	Experimental Models	Intervention	Results	References
Pancreatic cancer				
In vitro				
Thymoquinone and Gemcitabine	PANC-1, AsPC-1 and BxPC-3	0–50 (Thymoquinone) and 0–200 μmol/L (Gemcitabine) for 48 h	- Promotion of apoptosis - Inhibition of tumor growth	[113]
TQ analogs (TQ-2G, TQ-4A1 and TQ-5A1) and Gemcitabine or Oxaliplatin	MiaPaCa-2	10 (Thymoquinone analogs) and 0.5 μM (Gemcitabine) or 6.0 μg/mL (Oxaliplatin) for 84 h	- Enhancement of apoptosis	[67]
ATQTHB or ATQTFB analogs and Gemcitabine	MiaPaCa-2 cells	2.5 (analog) and 0.5 μM (Gemcitabine) for 24h and 72 h	- Enhancement of cytotoxic effect	[68]
Thymoquinone and Gemcitabine	MIA PaCa-2 and PANC-1 cells	25–36 μM for 48 h	- Inhibition of cancer cell proliferation - Inhibition of pyruvate kinase M2	[114]
In vivo				
Thymoquinone and Gemcitabine	BALB/c nude mice	1.0 mg/day (Thymoquinone) and 50 mg/kg 3 times/week (Gemcitabine),	- Reduction of tumor weight	[113]
Breast cancer				
In vitro				
Thymoquinone and Gemcitabine	MCF-7 cells and T47D	0.01 to 300 μM for 24, 48 and 72 h	- Induction of apoptosis and necrosis - Increase of autophagic cell death - Depletion of tumor associated - resistant stem cell fraction	[115]
Thymoquinone and Paclitaxel	4T1 cells	6.25, 12.5 and 25 (Thymoquinone) μM and 10 μg/mL (Paclitaxel) for 24 h	- Inhibition of cancer cell growth - Induction of apoptosis - Induction of cytotoxicity	[116]
Thymoquinone and Cisplatin or Docetaxel	MDAMB-468	0.5–2 μM Thymoquinone for 24 h and 72 h	- Increase of cytotoxicity - Reduction of Akt activation	[41]
In vivo				
Thymoquinone and Doxorubicin	MDA-MB-231 cell xenograft nude mice	4 mg/kg/6 days/week (Thymoquinone) and 2.5 mg/kg/once/per week	- Antitumor effect - Downregulation of antiapoptotic gene	[35]
Colon cancer				
In vivo				
Thymoquinone and Vitamin D3	azoxymethane treated rat	35 mg/kg/day, three days/week (Thymoquinone) and 500 IU/day, 3 days/week (Vitamin D3)	- Reduction of tumor growth - Decrease of large aberrant crypts foci - Increase of antitumorigenesis biomarker	[117]
Thymoquinone and 5-Fluorouracil	Azoxymethane treated male Wistar rats	35 mg/kg/d for 3 d/week (Thymoquinone) and 12 mg/kg/d for 4 days after 6 mg/kg/day for 5-Fluorouracil	- Reduction of tumor growth - Decrease of large aberrant crypts foci	[118]
Lung cancer				
In vitro				
Thymoquinone and Cisplatin	NCI-H460 and NCI-H146	80 and 100 μM (Thymoquinone) and 1.25, 2.5 and 5.0 (Cisplatin) for 24, 48 and 72 h	- Inhibition of cell proliferation	[61]
Thymoquinone and Cisplatin	LNM35	10 and 50 μM (Thymoquinone) and 10 μM (Cisplatin) for 24 h	- Inhibition of cellular viability	[119]
In vivo				
Thymoquinone and Cisplatin	Severe combined immunodeficiency mice	5 and 20 mg/kg/2days for 3 weeks (Thymoquinone) and 2.5 (Cisplatin) mg/kg/week for 3 weeks	- Decrease of tumor volume and weight	[61]
Leukemia				
In vitro				
Thymoquinone and Doxorubicin	HL-60 cells	5 μM for 24 h	- Increase anticancer effect	[120]
Thymoquinone and Doxorubicin	Jurkat cells	0–30 μm for 24, 48 and 72 h	- Inhibition of cell proliferation - Induction of apoptosis	[121]
Renal Cancer				
In vivo				
*N. sativa* oil and Cisplatin	male Wistar rats	2 mL/kg (Thymoquinone) and 3 mg/kg (Cisplatin) body wt for 20 days	- Improvement of nephropathy	[123]
Thymoquinone and Cisplatin	male Wistar rats	1.5 mg/kg (Thymoquinone) and 3 mg/kg body wt (Cisplatin) for 20 days	- Improvement of nephropathy	
Ovarian cancer				
In vitro				
Thymoquinone and Cisplatin or Oxaliplatin	A2780 and A2780 cisR cells	2.28–36.49 and 1.93- 30.83 μM (Thymoquinone) 0.26–4.09 and 1.66–26.52 μM (Cisplatin), 0.16–2.62 and 0.59–9.41 μM (Oxaliplatin) for 72 h	- Overcome of drug resistance	[124]
Thymoquinone and Cisplatin	SK-OV-3 cells	10, 15, 20 and 25 µmol/L(Thymoquinone) and 5, 10, 15 and 20 µmol/L (Cisplatin) for 24, 48, and 72 h	- Enhancement of apoptosis - Arrest of cell cycle in S phase	[125]
Thymoquinone and Cisplatin	Murine ID8-NGL cells	2.5, 5, 10,20, 25,50 μM (Thymoquinone) and 0.25, 0.5, 1, 2,2.5, 5 (Cisplatin) μM for 72 h	- Inhibition of tumor growth - Induction of apoptosis - Inhibition of cell viability - Increase of cytotoxicity	[95]
In vivo				
Thymoquinone and Cisplatin	ID8-NGL treated C57BL/6 mice	20 mg/kg/week for three times (Thymoquinone), 2 mg/kg /weekly (Cisplatin) for 30 days	- Decrease of overall tumor burden - Increase of DNA damage	[95]
Prostate cancer				
In vitro				
Thymoquinone and Zoledronic acid	PC-3 and DU-145 cells	55.3 and 51.0 μM (Thymoquinone) and 95.0, 52.9 μM (Zoledronic acid) for 24, 48 and 72 h	- Enhancement of cytotoxicity - Induction of apoptosis	[126]
Thymoquinone and Docetaxel	DU-145 cells	60 μM (Thymoquinone) and 0.1 and 10 nM (Docetaxel) for 24, 48 and 72 h	- Enhancement of cytotoxicity - Induction of apoptosis	[99]
Oral cancer				
In vitro				
Thymoquinone and Diosgenin	Human SCC A431, Hep2 and RPMI 2650 cells	10 µM (Thymoquinone) and 20 µM (Diosgenin) for 48 h	- Inhibition of cell proliferation - Induction of apoptosis	[94]
Thymoquinone and Cisplatin	UMSCC-14C cells	0.01–100 μM for 24, 48 and 72 h	- Induction of apoptosis - Inhibition of cell viability - Improvement of cytotoxic effect	[127]
Head and neck cancer				
In vitro				
Thymoquinone and radiation	SCC25 and CAL27 HNSCC cell lines	0–80 μM (Thymoquinone) and 2 Gy/min for 72 h	- Reduction of survival	[109]
